# 基于通过型固相萃取-超高效液相色谱-高分辨质谱同时测定杨梅中29种农药残留

**DOI:** 10.3724/SP.J.1123.2020.11011

**Published:** 2021-06-08

**Authors:** Shengdong PAN, Yanbo GUO, Li WANG, Dandan ZHANG

**Affiliations:** 宁波市疾病预防控制中心, 浙江省微量有毒化学物健康风险评估技术研究重点实验室, 浙江 宁波 315010; Key Laboratory of Health Risk Appraisal for Trace Toxic Chemicals of Zhejiang Province,Ningbo Municipal Center for Disease Control and Prevention, Ningbo 315010, China; 宁波市疾病预防控制中心, 浙江省微量有毒化学物健康风险评估技术研究重点实验室, 浙江 宁波 315010; Key Laboratory of Health Risk Appraisal for Trace Toxic Chemicals of Zhejiang Province,Ningbo Municipal Center for Disease Control and Prevention, Ningbo 315010, China; 宁波市疾病预防控制中心, 浙江省微量有毒化学物健康风险评估技术研究重点实验室, 浙江 宁波 315010; Key Laboratory of Health Risk Appraisal for Trace Toxic Chemicals of Zhejiang Province,Ningbo Municipal Center for Disease Control and Prevention, Ningbo 315010, China; 宁波市疾病预防控制中心, 浙江省微量有毒化学物健康风险评估技术研究重点实验室, 浙江 宁波 315010; Key Laboratory of Health Risk Appraisal for Trace Toxic Chemicals of Zhejiang Province,Ningbo Municipal Center for Disease Control and Prevention, Ningbo 315010, China

**Keywords:** 固相萃取, 超高效液相色谱-高分辨质谱, 农药残留, 杨梅, 基质效应, solid-phase extraction (SPE), ultra-performance liquid chromatography-high resolution mass spectrometry (UPLC-HRMS), pesticide residues, bayberry, matrix effect (ME)

## Abstract

建立了基于PRiME HLB通过型固相萃取净化-超高效液相色谱-高分辨质谱法(UPLC-HRMS)快速准确测定杨梅中29种常见农药残留的检测方法。杨梅样品经乙腈涡旋提取、盐析和PRiME HLB固相萃取净化后,以5 mmol/L乙酸铵水溶液和乙腈溶液作为流动相在Waters ACQUITY UPLC HSS T_3_色谱柱(100 mm×2.1 mm, 1.8 μm)上进行色谱分离,采用正离子电喷雾离子化模式(ESI^+^)和一级全扫描-数据依赖二级质谱扫描模式(Full mass-ddMS^2^),基质匹配外标法定量分析。该研究首先优化了液相色谱条件,重点考察了Waters ACQUITY UPLC HSS T_3_色谱柱和Waters ACQUITY UPLC BEH C_18_色谱柱对29种农药色谱行为的影响,结果表明Waters ACQUITY UPLC HSS T_3_色谱柱相比后者具有更强的色谱保留能力;流动相优化结果显示,相比于乙腈-甲酸水溶液体系和乙腈-甲酸-乙酸铵水溶液体系,乙腈-乙酸铵水溶液体系作为流动相时29种农药普遍具有更佳的色谱保留,部分农药的质谱响应有了显著的提高。此外,该研究通过考察3种不同净化方法的基质效应以优化杨梅中29种农药残留检测,实验结果表明,相比于GCB SPE和QuEChERS法两种净化方式,PRiME HLB法对于杨梅提取液具有较好的基质净化能力。在最佳实验条件下,29种农药在1.0~200.0 μg/L范围内呈现良好的线性关系(线性相关系数*R*^2^>0.999),方法检出限为2.0 μg/kg;低(6 μg/kg)、中(200 μg/kg)、高(400 μg/kg)3个加标水平下,29种农药的加标回收率为69.2%~135.6%,相对标准偏差为0.7%~14.6%。该方法具有快速、简便、灵敏和准确等优点,适用于理化实验室大批量样品的日常监测。

## Abstract (en)

A rapid and accurate analysis method based on PRiME HLB pass-through solid-phase extraction (SPE) and ultra-performance liquid chromatography-high resolution mass spectrometry (UPLC-HRMS) was developed for the determination of 29 pesticide residues in bayberry samples. The bayberry samples were first extracted using acetonitrile by vortexing; then, the extract solution was salted out and purified by PRiME HLB pass-through solid-phase extraction (SPE) cartridges. Chromatographic separation was subsequently carried out on a Waters ACQUITY UPLC HSS T_3_ column (100 mm×2.1 mm, 1.8 μm) using 5 mmol/L ammonium acetate in water and acetonitrile as the elution solvent. The electrospray ion source in positive (ESI^+^) mode and full mass-data-dependent MS^2^ (full mass-ddMS^2^) mode were used for quantification by the matrix-matched external standard method. The LC conditions were first optimized, and two analytical columns, Waters ACQUITY UPLC HSS T_3_ and Waters ACQUITY UPLC BEH C_18_, were investigated for the 29 pesticides. The results indicated that the Waters ACQUITY UPLC HSS T_3_ column showed better chromatographic retention. Moreover, composites of the mobile phase were also studied. Compared to the acetonitrile-formic acid aqueous solution system and acetonitrile-formic acid-ammonium acetate aqueous solution system, the acetonitrile-ammonium acetate aqueous solution system used as the mobile phase exhibited much better chromatographic behavior for most of the 29 pesticides. In particular, the MS responses of some of the target pesticides were significantly improved when using the ammonium acetate-acetonitrile system as the mobile phase. In addition, the sample pretreatment conditions for the 29 pesticides in bayberry samples were systematically optimized. The matrix effect (ME) for three different types of purification methods were applied to evaluate the purification efficiency for the 29 pesticides in the bayberry samples. The following results were obtained from the post-spiking experiments: (1) For graphitized carbon (GCB) SPE, the post-spiking recoveries of 29 pesticides in the bayberry samples were generally low, less than 60%. (2) For the QuEChERS method, the recoveries of most target pesticides improved. The pesticide ratio with recoveries ranging from 70% to 120% was found to be 41%; however, the pesticide ratio with recoveries of less than 60% was still high (35%). (3) For the PRiME HLB-based pretreatment method, the recoveries of the 29 pesticides obviously improved. The pesticide ratio with recoveries between 70% and 120% was up to 76%, while the pesticide ratios were only 14% and 10% for post-spiking recoveries of 60%-70% and >120%, respectively. Meanwhile, the recoveries of all 29 pesticides were found to be more than 60%. Therefore, the PRiME HLB-based method was better than the GCB SPE and QuEChERS methods for pretreatment of the 29 pesticides in the bayberry samples. In addition, the PRiME HLB-based pretreatment process does not require tedious operation processes such as activation, balance, and elution, and thus, the sample pretreatment time is greatly shortened.

Under the optimal conditions, the 29 target pesticides showed good linearity in the range of 1.0-200.0 μg/L, with correlation coefficients (*R*^2^) higher than 0.999. The limits of detection (LODs) were 2.0 μg/kg for the 29 target pesticides. The recoveries of the pesticides spiked in the bayberry samples were in the range of 69.2%-135.6% at 6, 200, and 400 μg/kg, respectively, while the relative standard deviations (RSDs) in the range of 0.7%-14.6%. The proposed method based on PRiME HLB-pass through SPE-UPLC-HRMS was adopted to determine these 29 pesticides in 30 bayberry samples purchased from local and online markets. According to the results, pesticides such as methamidamine, difenoconazole, and tebuconazole were frequently detected in the bayberry samples. However, the maximum residue limits (MRLs) of methamidamine, difenoconazole, and tebuconazole in bayberry samples were not provided in GB 2763-2019. In summary, the developed method is fast, simple, sensitive, and accurate, and it can be applied for daily monitoring of pesticides in bayberry samples.

杨梅在生长过程极易受到褐斑病、白腐病、松毛虫、卷叶蛾、介壳虫和果蝇等病虫害影响,需施用大量农药加以防治^[1]^。目前,杨梅实际登记可使用的农药只有11种,但生产过程中存在农药登记种类少、实际使用种类多的问题^[2]^,从而导致无法精准监测和掌握杨梅中实际使用的农药残留情况。此外,由于杨梅属于小宗农作物,其生产技术标准还不够完善,缺乏相应的指导标准,客观上造成乱用药、过度用药的现象。目前GB 2763-2019只规定了杨梅中48种农药残留限量标准^[3]^,诸如咪鲜胺、苯醚甲环唑、戊唑醇、多菌灵等在杨梅实际生产过程中使用率高的农药未见有限量规定。综上所述,标准缺失和生产过程中操作不当等因素导致杨梅中农药残留与超标的问题,可能造成杨梅食用过程中存在安全隐患。因此,开发快速、简便、准确的检测杨梅中多组分农药残留的分析方法具有十分重要的现实意义。

目前,多组分农药残留分析已成为理化检验的新趋势与新热点,常用的农药残留检测方法主要包括气相色谱法(GC)^[4,5]^、气相色谱-质谱联用法(GC-MS)^[6,7,8,9]^、液相色谱法^[10,11]^(LC)和液相色谱-质谱联用法(LC-MS)等^[12,13,14,15,16]^。其中GC和GC-MS主要针对低沸点农药的检测,对于难挥发的农药需要进行衍生化反应后方能测定,增加了操作过程的繁琐性,同时衍生化过程的不确定性给多组分农药残留检测带来了极大挑战。LC虽能准确测定果蔬中农药残留,但受限于紫外和荧光检测器的灵敏度和定性能力,LC常常得到假阳性的检测结果。随着液相色谱-质谱联用仪在基层实验室的逐渐普及,凭借其快速、高灵敏、兼容性好等优点,LC-MS已成为果蔬中多组分农药残留检测过程中较优的选择。随着LC-HRMS技术的日趋成熟,利用精确相对分子质量定性与定量所体现的优势为食品中有毒有害污染物的快速测定提供了有效的解决方案。尤其对于低相对分子质量或较强极性的农药残留检测,LC-HRMS能弥补液相色谱-串联质谱(LC-MS/MS)定性能力差的不足。另外,在LC-MS检测过程中基质干扰效应是不可忽视的重要因素,处理不当会严重影响检测结果的准确性,需要选择合适的样品前处理技术对果蔬样品提取物进行有效的净化,进而达到准确测定的目的。目前,适合于大批量果蔬样品中农药残留检测的样品前处理技术主要包括QuEChERS法^[8,17]^和固相萃取法(SPE)^[15,18]^。其中,QuEChERS法是基于分散固相萃取技术发展起来的一种兼具萃取与净化功能为一体的新型样品前处理方法。该方法经常使用C_18_粉末、PSA粉末和石墨化炭黑等作为净化材料,但由于上述净化材料特异性差,尤其在分子极性差异性大的多组分农药的同时净化过程中,常常会造成部分农药的损失,影响检测结果的准确性。SPE法是一种有效的样品前处理技术,目前商品化的SPE小柱种类繁多,如C_18_、HLB、MCX、MAX等不同类型的固相萃取柱,可根据不同化合物的结构特点选择合适的SPE小柱。PRiME HLB固相萃取柱是Waters公司在HLB固相萃取柱技术基础之上开发的新一代产品,能高效吸附与去除样品中蛋白质与磷脂类物质,可有效降低LC-MS检测过程中的基质干扰效应^[19]^。然而,目前将PRiME HLB固相萃取柱主要用于动物源性食品中药物残留检测过程中的样品前处理^[20,21,22]^,显示出优良的净化性能。经文献检索结果表明,但目前鲜有文献报道PRiME HLB固相萃取柱在植物性样品中农药残留检测中的应用,然而植物性样品中同样存在影响基质效应的磷脂类物质,因此,PRiME HLB固相萃取柱有望在杨梅农药残留检测过程中发挥重要作用。

由于杨梅属于小宗水果,目前尚无专门针对杨梅中农药残留检测的国家标准方法,关于杨梅中多组分农药残留的检测技术研究的文献数量也比较少,且许多文献检测的农药种类较少^[1,23]^,或主要关于有机磷和菊酯类农药残留的检测^[8]^,对于高使用频率的杀菌剂等农药残留检测的报道相对较少。基于上述存在的问题,本文通过对杨梅中3-羟基克百威等29种农药残留检测方法的系统研究,考察了3种净化方法对基质效应和回收率的影响,筛选出最优的样品前处理方案,建立了基于PRiME HLB通过型固相萃取-超高效液相色谱-高分辨质谱(SPE-UPLC-HRMS)同时测定杨梅中29种农药残留的检测方法,可用于实验室大批量杨梅样品中农药残留的日常监测。

## 1 实验部分

### 1.1 仪器与试剂

Waters UPLC I Class型超高效液相色谱仪(美国Waters公司); Q-Exactive Orbitrap型高分辨质谱仪(美国Thermo-Fisher公司)和Trace Finder 3.3数据处理系统;Sigma低温高速离心机(德国Sigma公司); 20位固相萃取装置(美国Agilent公司)。

乙腈和甲酸(LC-MS级)均购自美国Thermo-Fisher公司;乙酸铵(HPLC级)购自德国Merck公司;有证标准品3-羟基克百威、三唑酮、丙溴磷、乐果、乙酰甲胺磷、仲丁威、克百威、吡虫啉、啶虫脒、嘧霉胺、多菌灵、异丙威、戊唑醇、氧化乐果、氯虫苯甲酰胺、灭蝇胺、烯酰吗啉、甲基硫菌灵、甲拌磷砜、甲拌磷亚砜、甲萘威、甲霜灵和苯醚甲环唑均购自德国Dr. Ehrenstorfer公司,纯度均>95%;有证标准溶液克百威(1000 mg/L)、涕灭威(100 mg/L)、涕灭威亚砜(100 mg/L)、涕灭威砜(1000 mg/L)、甲拌磷(100 mg/L)和甲胺磷(100 mg/L)购自北京振翔科技有限公司,表1列举了29种农药的有关信息;PRiME HLB固相萃取柱(200 mg/6 mL)购自美国Waters公司;ProElut CAPR Glass (GCB)固相萃取柱(500 mg/6 mL)购自迪马科技;QuEChERS净化管(15 mL)购自美国安捷伦公司;Waters ACQUITY UPLC HSS T_3_色谱柱(100 mm×2.1 mm, 1.8 μm)和Waters ACQUITY UPLC BEH C_18_色谱柱(100 mm×2.1 mm, 1.7 μm)购自美国Waters公司。

**表 1:** 29种农药分子式、离子加合方式、精确质荷比和保留时间

No.	Compound	Molecular formula	Adduct ion	Exact m/z	Retention time/min
1	3-hydroxygram budweiser (3-羟基克百威)	C_12_H_15_NO_4_	[M+H]^+^	238.10738	3.37
2	ketotriazole (三唑酮)	C_14_H_16_ClN_3_O_2_	[M+H]^+^	294.10038	5.66
3	brominated phosphorus (丙溴磷)	C_11_H_15_BrClO_3_PS	[M+H]^+^	372.94242	6.70
4	rogor (乐果)	C_5_H_12_NO_3_PS_2_	[M+H]^+^	230.00690	3.72
5	orthene (乙酰甲胺磷)	C_4_H_10_NO_3_PS	[M+H]^+^	184.01918	1.06
6	fenobucarb (仲丁威)	C_12_H_17_NO_2_	[M+H]^+^	208.13321	5.46
7	carbofuran (克百威)	C_12_H_15_NO_3_	[M+H]^+^	222.11247	4.81
8	imidacloprid (吡虫啉)	C_9_H_10_ClN_5_O_2_	[M+H]^+^	256.05958	3.53
9	methamidamine (咪鲜胺)	C_15_H_16_Cl_3_N_3_O_2_	[M+H]^+^	376.03809	6.07
10	acetamiprid (啶虫脒)	C_10_H_11_ClN_4_	[M+H]^+^	223.07450	3.80
11	pyrimamine (嘧霉胺)	C_12_H_13_N_3_	[M+H]^+^	200.11822	5.51
12	carbendazim (多菌灵)	C_9_H_9_N_3_O_2_	[M+H]^+^	192.07675	3.37
13	isoprocarb (异丙威)	C_11_H_15_NO_2_	[M+H]^+^	194.11756	5.15
14	tebuconazole (戊唑醇)	C_16_H_22_ClN_3_O	[M+H]^+^	308.15242	5.77
15	folimat (氧化乐果)	C_5_H_12_NO_4_PS	[M+H]^+^	214.02974	1.13
16	chlorobenzamide (氯虫苯甲酰胺)	C_18_H_14_BrCl_2_N_5_O_2_	[M+H]^+^	481.97807	5.32
17	aldicarb (涕灭威)	C_7_H_14_N_2_O_2_S	[M+Na]^+^	213.06682	4.32
18	aldicarb sulfoxide (涕灭威亚砜)	C_7_H_14_N_2_O_3_S	[M+H]^+^	207.07979	1.20
19	aldicarb sulfone (涕灭威砜)	C_7_H_14_N_2_O_4_S	[M+NH_4_]^+^	240.10125	1.68
20	tetramethylamine (灭蝇胺)	C_6_H_10_N_6_	[M+H]^+^	167.10397	0.87
21	dimethomorph (烯酰吗啉)	C_21_H_22_ClNO_4_	[M+H]^+^	388.13101	5.35
22	thiophonate-methyl (甲基硫菌灵)	C_12_H_14_N_4_O_4_S_2_	[M+H]^+^	343.05292	4.62
23	phorate (甲拌磷)	C_7_H_17_O_2_PS_3_	[M+H]^+^	261.02010	6.47
24	phosphate sulfone (甲拌磷砜)	C_7_H_17_O_4_PS_3_	[M+H]^+^	293.00992	5.37
25	phorate sulfoxide (甲拌磷亚砜)	C_7_H_17_O_3_PS_3_	[M+H]^+^	277.01502	5.37
26	methamidophos (甲胺磷)	C_2_H_8_NO_2_PS	[M+H]^+^	142.00861	0.97
27	carbaryl (甲萘威)	C_12_H_11_NO_2_	[M+H]^+^	202.08626	4.91
28	metalaxyl (甲霜灵)	C_15_H_21_NO_4_	[M+H]^+^	280.15433	5.07
29	difenoconazole (苯醚甲环唑)	C_19_H_17_Cl_2_N_3_O_3_	[M+H]^+^	406.07197	6.10

30份杨梅样品购自菜场、超市和天猫超市等。

### 1.2 样品前处理

将采集的杨梅样品采用搅拌机粉碎混匀,储存于50 mL塑料离心管内,-20 ℃下保存,待测。

准确称取经混匀的杨梅样品5.0 g(精确至0.01 g)于50 mL离心管中,加入10 mL乙腈涡旋提取20 min,然后分别加入1 g氯化钠和4 g无水硫酸镁(盐析), 8000 r/min离心3 min。取上清液5 mL,采用PRiME HLB固相萃取柱净化,收集后约2 mL流出液,准确吸取0.2 mL净化液于1 mL容量瓶中,用纯水稀释定容至刻度,过0.22 μm聚四氟乙烯滤膜,UPLC-HRMS进样分析。

### 1.3 色谱-质谱条件

色谱条件 色谱柱为Waters ACQUITY UPLC HSS T3色谱柱(100 mm×2.1 mm, 1.8 μm);流动相:A相,5 mmol/L乙酸铵溶液;B相,乙腈;梯度洗脱:0~2.00 min, 20%B; 2.00~5.00 min, 20%B~80%B; 5.00~8.00 min, 80%B; 8.00~8.01 min, 80%B~20%B; 8.01~10.00 min, 20%B;流速0.3 mL/min;进样量5 μL;柱温40 ℃。

质谱条件 离子源:电喷雾正离子模式(ESI^+^);离子传输管温度:320 ℃;定量检测方式:一级全扫描-数据依赖二级质谱扫描模式(Full mass-ddMS^2^);分辨率:全扫(Full Mass)70000,二级质谱扫描(MS/MS)17500;隔离窗口(isolation window): *m/z* 1.0;电喷雾电压:3500 V;鞘气压力:275.8 kPa;辅助气速率:180 L/h;反吹气压力:13.8 kPa;辅助气加热温度:300 ℃;射频棱镜电压(S-lens RF level): 50%。

### 1.4 标准溶液的配制

1.0 g/L农药单标溶液:分别准确称取10.0 mg固体标准物质于10.0 mL容量瓶中,用乙腈溶解、稀释、定容至刻度,混匀。

10 mg/L 29种混合标准溶液:分别准确吸取适量29种农药单标溶液于10 mL容量瓶中,用乙腈稀释并定容至刻度,混匀。

系列标准溶液:准确吸取适量10 mg/L 29种混合标准溶液于1 mL容量瓶中,采用20%(v/v)乙腈水溶液稀释并定容至刻度,配制成1.0、5.0、10.0、100.0和200.0 μg/L的农药标准系列。

基质匹配工作曲线:准确称取混匀的空白杨梅样品5.0 g(精确至0.01 g)于50 mL离心管中,加入10 mL乙腈涡旋提取20 min,然后分别加入1 g氯化钠和4 g无水硫酸镁(盐析), 8000 r/min离心3 min。取上清液5 mL,采用PRiME HLB固相萃取柱净化,收集后约2 mL流出液,准确吸取0.2 mL净化液于1 mL容量瓶中,然后分别加入适量10 mg/L 29种混合标准溶液,用纯水稀释并定容至刻度,配制成1.0、5.0、10.0、100.0和200.0 μg/L系列标准溶液。

## 2 结果与讨论

### 2.1 液相色谱条件优化

分别选择乙腈-甲酸水溶液体系、乙腈-乙酸铵-甲酸水溶液体系和乙腈-乙酸铵水溶液体系作为流动相,比较其对29种农药在Waters ACQUITY UPLC HSS T_3_色谱柱上的色谱行为影响,结果(以多菌灵为例)见图1。实验结果表明,当采用乙腈-甲酸水溶液体系作为流动相时,虽然多菌灵的色谱峰形对称,但在色谱柱上的保留较差(*t*_R_=1.29 min)(见图1a);当采用乙腈-甲酸-乙酸铵水溶液体系时,色谱保留行为稍有改善,但仍然不十分理想(*t*_R_=1.63 min)(见图1b);当采用乙腈-乙酸铵水溶液体系作为流动相时,多菌灵色谱峰形理想,且色谱保留有了明显改善(*t*_R_=3.43 min)(见图1c)。可能的原因是多菌灵分子极性较大,其结构中含有苯并咪唑基团和酰胺基团,在反相色谱柱上保留较差,当流动相体系中含有甲酸组分时,多菌灵分子的氮原子容易发生质子化作用形成正电荷,进一步增强了分子极性,因此多菌灵在乙腈-甲酸水溶液体系和乙腈-乙酸铵-甲酸-水溶液体系下色谱保留较差。同时,对于多菌灵而言,随着色谱保留时间的延长,质谱响应逐渐增强,即在乙腈-乙酸铵水溶液体系下质谱信号最强,乙腈-甲酸-乙酸铵水溶液体系下次之,乙腈-甲酸水溶液体系下质谱响应最弱。可能的原因是体系中大多数杂质在*t*_R_=1 min左右流出,能干扰该时间出峰的多菌灵的质谱离子化过程,从而影响其质谱响应,而乙腈-乙酸铵水溶液体系下多菌灵的保留时间较靠后(*t*_R_=3.43 min),此时共流出的干扰物相对较少,质谱干扰少,质谱信号强度较强。其他28种农药大多有类似多菌灵的色谱现象,因此后续的实验均采用乙腈-乙酸铵水溶液体系作为流动相。

**图 1:**
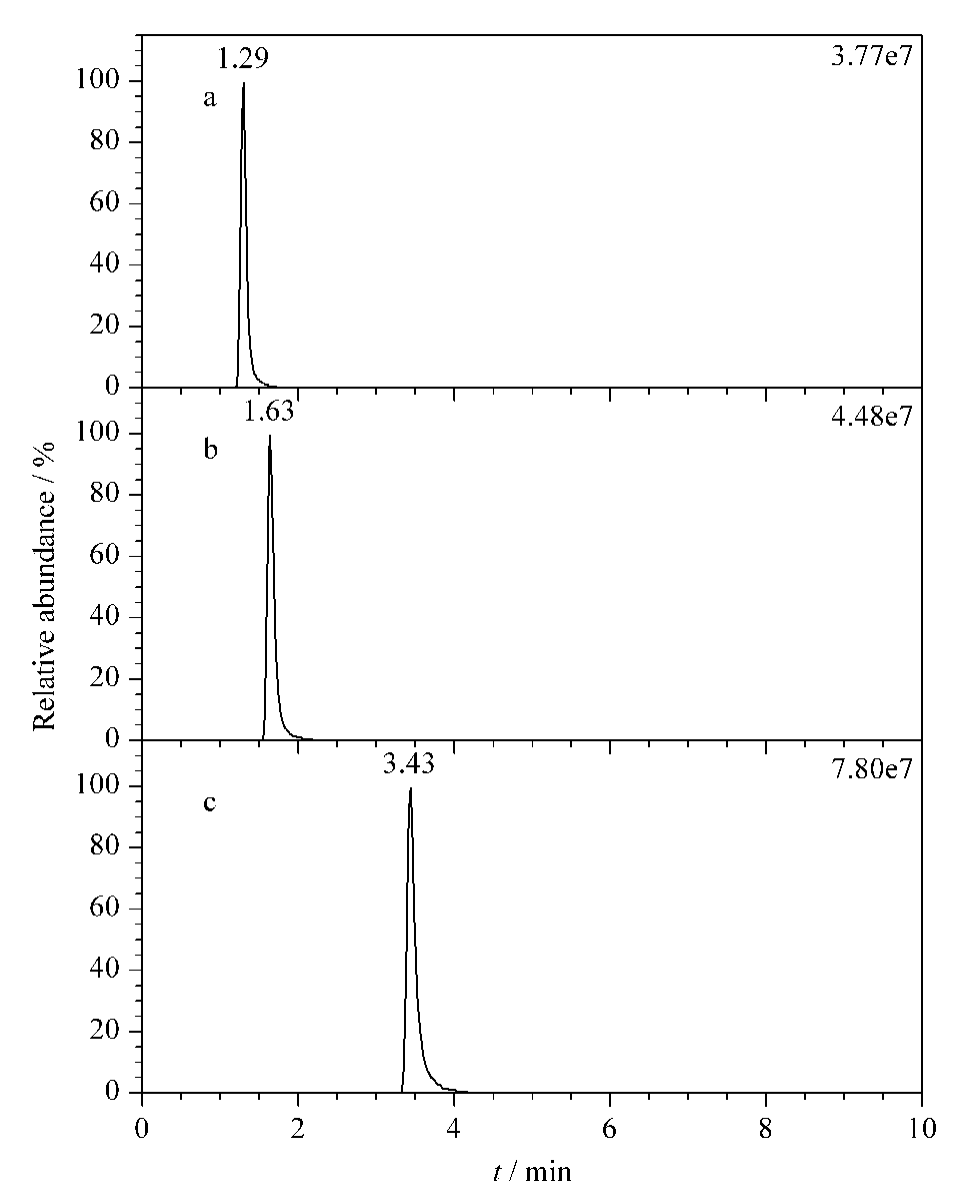
不同流动相条件下多菌灵的色谱行为

本研究考察了29种农药在Waters ACQUITY UPLC BEH C_18_和Waters ACQUITY UPLC HSS T_3_两款色谱柱上的色谱行为。实验结果表明29种农药在两款色谱柱上的色谱峰对称性好、峰形窄。然而,大多数农药在Waters ACQUITY UPLC HSS T_3_上的保留时间比在Waters ACQUITY UPLC BEH C_18_上多2~3 min。

如图2所示,嘧霉胺和戊唑醇在Waters ACQUITY UPLC HSS T_3_色谱柱上的色谱保留均优于Waters ACQUITY UPLC BEH C_18_色谱柱,且质谱信号也明显强于后者。可能的原因是:一方面,嘧霉胺和戊唑醇的分子结构中含有氨基或羟基等亲水基团,分子极性较强,而T_3_色谱柱对极性化合物的保留能力优于常规C_18_色谱柱;另一方面,色谱保留时间的延长有利于目标分析物与极性杂质的有效分离,基质干扰效应小,使得嘧霉胺和戊唑醇在T_3_色谱柱上的质谱信号强度较高。综上所述,Waters ACQUITY UPLC HSS T_3_色谱柱更适合于29种农药的色谱分离。

**图 2:**
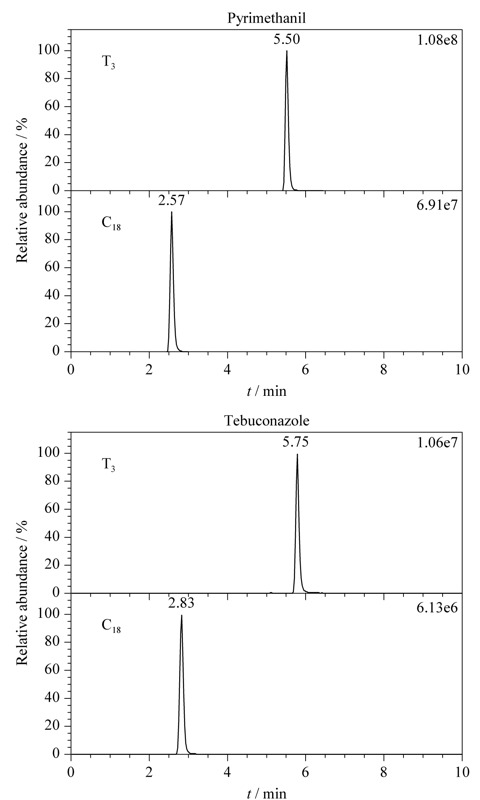
两种农药在不同色谱柱上的保留行为

### 2.2 固相萃取柱的选择与评价

根据文献^[2,8]^,乙腈能较好地提取杨梅中常见的农药残留,本研究不再进一步优化提取溶剂,后续实验均采用乙腈作为提取溶剂。本工作重点考察了3种净化方式(PRiME HLB柱、GCB SPE柱和QuEChERS净化管)对杨梅中29种农药残留检测的净化能力。具体实验过程为:准确称取3份5.0 g杨梅阴性样品,加入10 mL乙腈,涡旋提取20 min,然后分别加入1 g氯化钠和5 g无水硫酸镁,继续涡旋1 min,然后于8000 r/min离心3 min。取上清液5 mL,然后加入10 mg/L 29种农药混合标准溶液25 μL(后加标),分别采用PRiME HLB柱、GCB SPE柱和QuEChERS净化管净化。然后分别取3种净化液0.2 mL,用水定容至1 mL, 0.22 μm滤膜过滤,UPLC-HRMS进样分析,比较3种净化方式下杨梅中29种农药的后加标回收率,结果见图3和图4。

**图 3:**
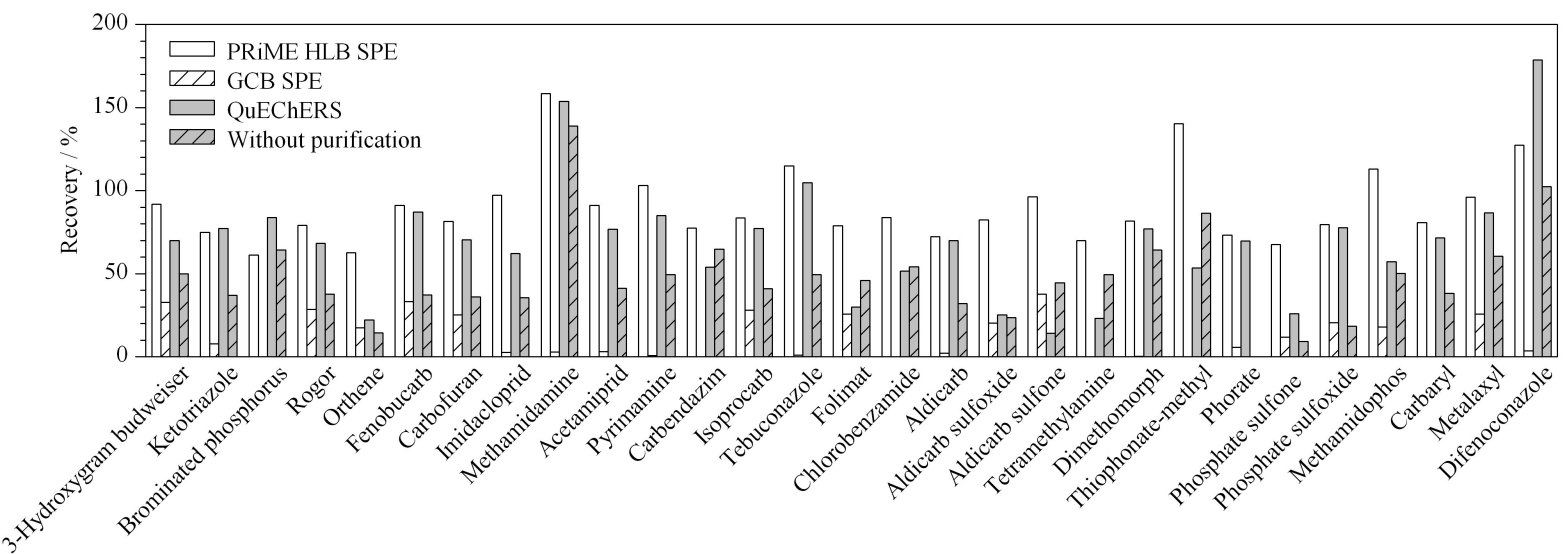
不同净化方法的净化性能对比

**图 4:**
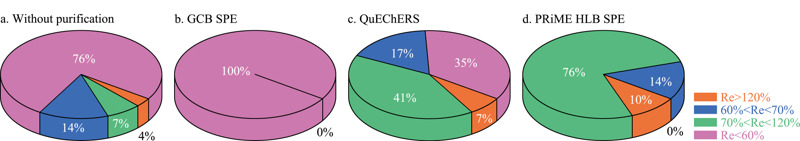
不同净化方法的后加标回收率(Re)占比

由图3可知,对于未经净化处理的杨梅样品,其加标回收率普遍不理想,其中回收率位于70%~120%区间的农药仅占比7.0%,回收率低于60%的农药占比最大,为76%(见图4a);经GCB SPE法净化后,29种农药的回收率普遍降低,均小于60%(图4b),可能的原因是石墨化炭黑填料对大多数农药成分均有一定的吸附能力,从而导致在样品净化过程中造成目标分析物的损失;杨梅提取液经QuEChERS净化管净化后,29种农药的回收率有了明显提升,其中回收率位于70%~120%区间的农药占比增加至41%,然而,该净化方法尚存在明显缺陷,回收率低于60%的农药仍占比35%(见图4c),无法满足准确定量的要求;杨梅提取液经PRiME HLB法净化后,29种农药的回收率有了大幅的改善,其中回收率位于70%~120%区间的农药占比为76%,位于60%~70%区间的农药占比14%,其余10%的农药回收率大于120%,而该净化方法对于回收率小于60%的农药已清零;由此可见,相比于GCB SPE和QuEChERS法,PRiME HLB法更适合于杨梅中农药残留检测的样品前处理过程。可能的原因是,PRiME HLB固相萃取小柱能更好地去除杨梅样品中的磷脂和蛋白质等杂质,能有效降低这些杂质对农药残留检测的干扰。此外,基于PRiME HLB的净化过程不需要活化、淋洗和洗脱等繁琐的操作过程,可大大缩短样品前处理时间。因此,后续实验均采用PRiME HLB法作为杨梅中农药残留检测的前处理方法。

### 2.3 方法评价

2.3.1 线性方程和基质效应评价

基质效应是由于离子化过程中样品中杂质组分与目标分析物相互竞争所致,从而导致目标分析物的质谱响应受到影响。如果目标分析物的质谱信号减弱,为基质抑制效应,质谱信号增强即为基质增强效应。

本研究分别比较了溶剂工作曲线、3种净化方式基质匹配工作曲线(PRiME HLB净化、GCB SPE净化和QuEChERS净化)和未净化的基质匹配工作曲线,结果表明29种农药在5种方式下在1~200 μg/L范围内均呈现良好的线性关系,线性相关系数(*R*^2^)>0.999。采用公式*η*=(基质匹配标准曲线斜率-溶剂标准曲线斜率)/溶剂标准曲线斜率评价杨梅中29种农药的基质效应^[24,25]^, *η*值越大,说明基质效应越大,且*η*值正负分别代表是基质增强和基质抑制作用。由图5可以看出,采用不同的净化方式,杨梅中不同农药的基质效应差异性较大。如,杨梅中的3-羟基克百威在未净化条件及3种净化方式下均具有较弱的基质干扰效应;三唑酮、啶虫脒和戊唑醇在未净化条件下均具有较强的基质抑制效应(*η*值为-33.3%~-50.0%),而经过3种方式净化后基质干扰效应消除(*η*值为0);乙酰甲胺磷、克百威、氧化乐果、涕灭威亚砜、涕灭威砜、灭蝇胺和烯酰吗啉采用GCB SPE或QuEChERS两种净化方式不能有效消除或减少基质干扰效应,甚至比未净化条件下基质抑制效应更强。

**图 5:**
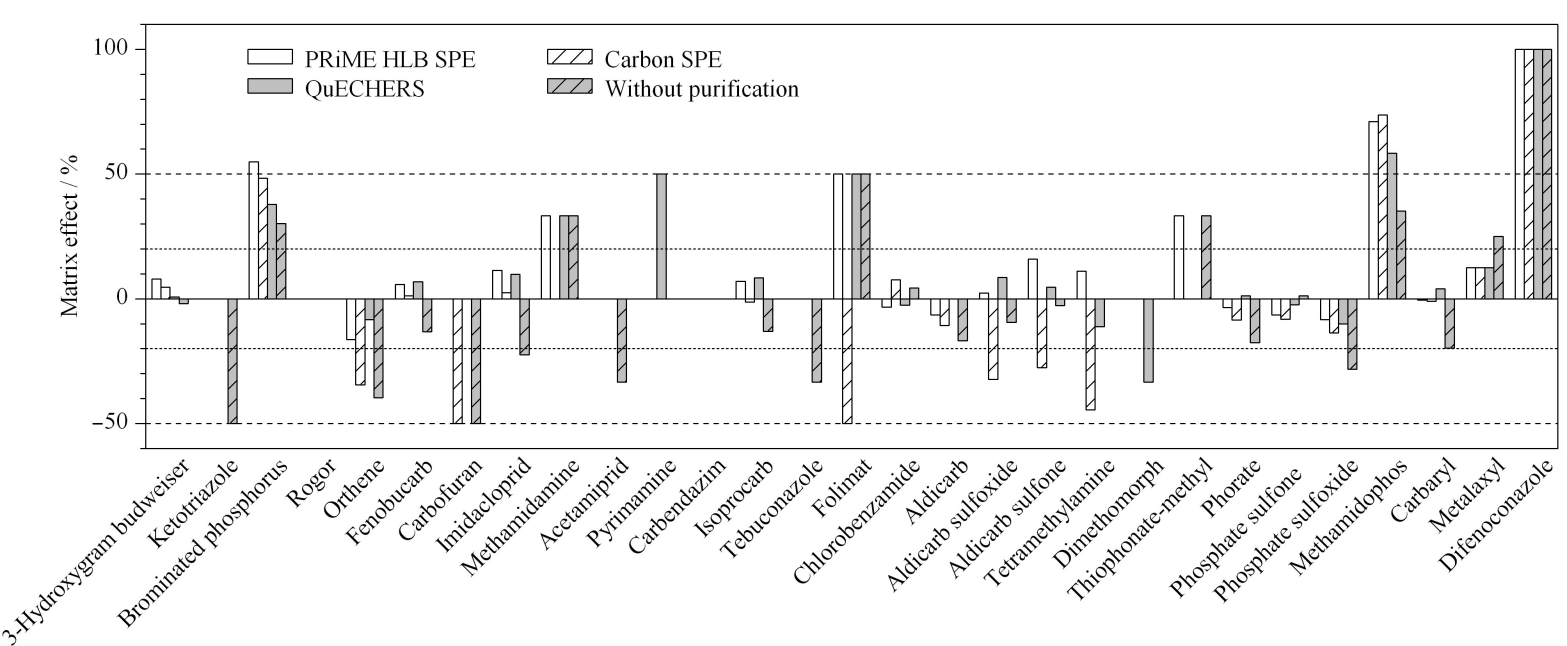
杨梅中29种农药在不同净化方式下的基质效应

为进一步评估不同净化方式对杨梅中29种农药的基质效应影响,本文根据文献^[26]^对不同农药的基质效应强度进行分级讨论,即当|*η*|<20%时,为弱基质效应,可忽略不计,采用纯溶剂配制标准工作曲线进行定量分析即可;当20% ≤ |*η*| ≤50%和|*η*|>50%,分别为中等基质效应和强基质效应,需要使用基质匹配工作曲线进行定量分析。根据该原则统计分析了采用3种净化方法时杨梅中29种农药的基质效应强弱情况,结果见图6。由图6可知,对于未经净化的杨梅样品,弱、中、强3个强度等级基质效应的占比分别为52%、34%和14%;经PRiME HLB SPE、GCB SPE和QuEChERS法3种净化方式处理以后,弱基质效应的比例均有了明显增加,分别增加至80%、76%和69%,同时中等强度基质效应的比例有所降低;但经GCB SPE和QuEChERS法两种净化方式处理的杨梅样品,强基质效应的农药比例未见变化,仍为14%,而经PRiME HLB SPE净化后强基质效应的农药比例降低至10%。与此同时,由图5可知,采用PRiME HLB SPE净化方法结果中所有中、强等级基质效应的农药均为正效应,即均为基质增强效应,可通过基质匹配工作曲线获得准确的定量结果,且基质增强效应可提高方法灵敏度;对于未净化和GCB SPE法,中、强等级基质效应农药中存在不同比例的基质抑制效应,即使采用基质匹配工作曲线法可以定量测定,但由于基质抑制效应的存在,检测灵敏度将受到较大的影响。

综上所述,采用PRiME HLB SPE净化方法可有效减弱基质抑制效应,采用基质匹配工作曲线能有效消除基质效应对检测结果的影响。

**图 6:**
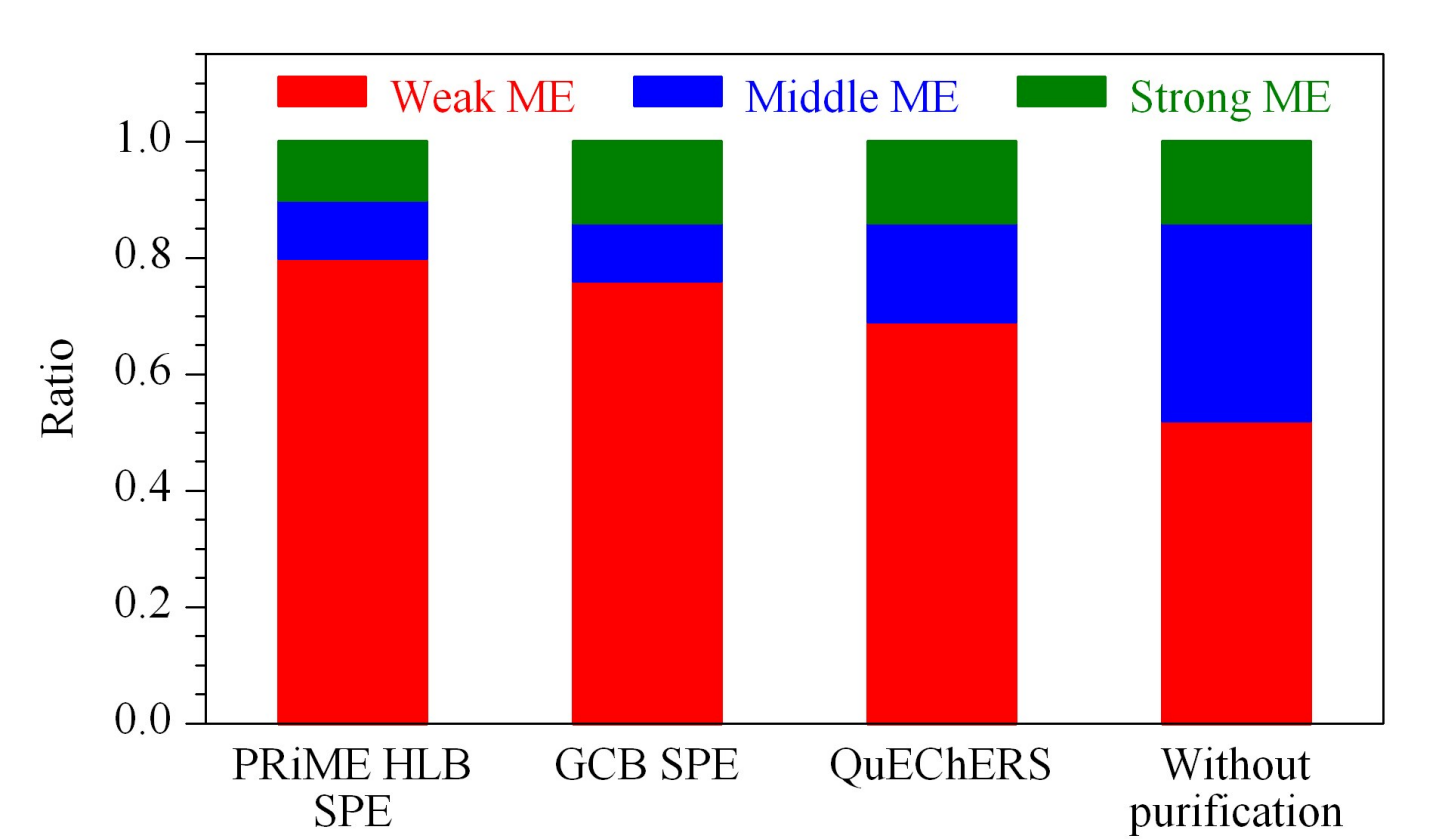
不同净化方式下农药基质效应强度等级分布比例

2.3.2 方法准确度、精密度、检出限和定量限

分别称取5.0 g杨梅空白样品,然后加入适量标准混合溶液,配制成低、中、高3个水平(6、200、400 μg/kg)的加标样品,按1.2节进行样品前处理,然后采用UPLC-HRMS检测,每个加标水平测定6次,结果见表2。由表2可知,29种农药在低、中、高3个加标水平下的回收率分别为73.8%~132.4%、69.2%~129.4%和73.3%~135.6%,RSD为0.7%~14.6%,能满足实验室日常监测的要求。其中仲丁威和低浓度异丙威的加标回收率相对较高(~130%),可能的原因是两者结构相似,杨梅提取液中仍存在能影响仲丁威和异丙威离子化效率的杂质难以去除。采用系列低浓度加标方式获得杨梅中29种农药的方法检出限(*S/N*≥3)为2.0 μg/kg,方法定量限(*S/N*≥10)为6.0 μg/kg。

**表 2:** 空白杨梅样品中29种农药的加标回收率和精密度(*n*=6)

Compound	Spiked levels							
	6 μg/kg			200 μg/kg			400 μg/kg	
	Recovery/%	RSD/%		Recovery/%	RSD/%		Recovery/%	RSD/%
3-Hydroxygram budweiser	86.2	2.3		97.3	1.7		94.2	1.1
Ketotriazole	90.1	3.6		93.8	3.4		105.2	2.0
Brominated phosphorus	88.2	5.7		96.7	2.0		87.4	2.0
Rogor	93.2	2.3		92.3	2.0		89.6	1.6
Orthene	79.2	8.9		82.1	2.9		82.9	2.2
Fenobucarb	131.5	5.1		129.4	3.6		135.6	4.4
Carbofuran	95.9	4.5		97.0	1.8		94.7	1.3
Imidacloprid	108.4	6.8		113.1	3.8		109.7	1.9
Methamidamine	81.8	6.1		85.2	2.8		75.7	1.4
Acetamiprid	112.5	2.1		104.6	1.7		96.3	1.0
Pyrimamine	121.3	12.8		110.1	10.6		101.8	3.0
Carbendazim	79.5	3.2		83.1	1.4		76.4	1.5
Isoprocarb	132.4	4.2		113.2	3.7		114.8	1.1
Tebuconazole	100.6	14.6		102.0	9.8		93.6	1.7
Folimat	73.8	3.2		81.9	1.4		76.3	0.9
Chlorobenzamide	98.5	5.3		90.8	4.7		85.6	2.1
Aldicarb	72.8	5.6		78.9	4.2		82.5	2.5
Aldicarb sulfoxide	79.3	8.7		69.2	1.2		73.3	1.8
Aldicarb sulfone	85.6	5.1		88.3	2.5		83.5	1.0
Tetramethylamine	74.2	3.5		82.5	1.8		76.4	0.7
Dimethomorph	99.3	8.4		91.7	7.3		82.8	1.3
Thiophonate-methyl	116.9	3.8		112.8	1.6		104.2	2.2
Phorate	70.6	9.2		81.3	6.1		77.6	5.2
Phorate sulfone	89.3	10.1		101.0	2.9		103.6	2.3
Phosphate sulfoxide	83.2	6.7		79.9	4.4		88.6	3.2
Methamidophos	90.6	4.8		111.4	2.9		108.6	0.8
Carbaryl	123.8	7.2		116.8	2.8		100.7	2.4
Metalaxyl	105.5	2.3		100.9	2.2		91.8	2.7
Difenoconazole	78.2	10.3		105.3	4.3		89.8	1.5

2.3.3 实际样品分析

应用本研究建立的方法对市售30份杨梅样品中的29种农药残留进行检测,结果表明杨梅中咪鲜胺、苯醚甲环唑和戊唑醇等农药有不同程度的检出,三者的检出量分别为0.0108~0.480 mg/kg、0.0136~0.241 mg/kg和0.0195~0.300 mg/kg,但GB 2763-2019未对上述3种农药的限量标准值有所规定,因此亟须相关行政部门及时修订农药限量标准。图7为1份典型性杨梅样品中3种农药残留的SIM图谱。

**图 7:**
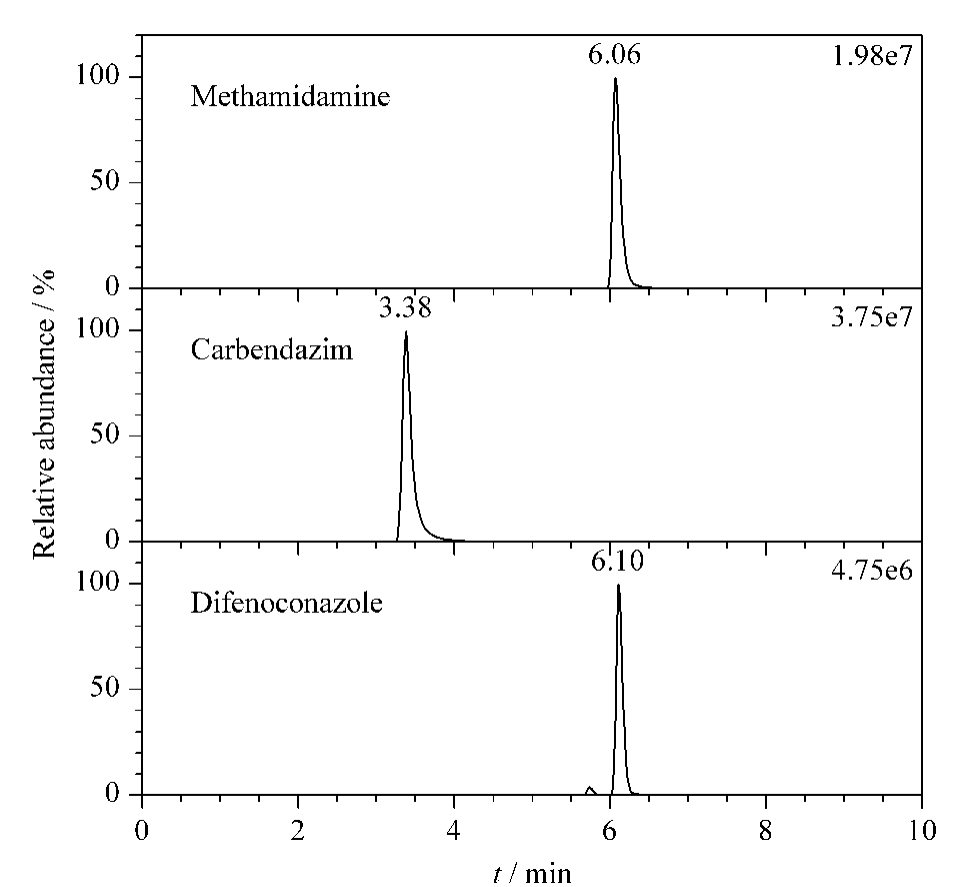
1份杨梅样品中3种农药的SIM色谱图

## 3 结论

本文通过对比3种净化方法在29种农药残留检测过程中的基质效应情况,最终优选了PRiME HLB作为杨梅样品的前处理方法,建立了基于PRiME HLB通过型固相萃取-超高效液相色谱-高分辨质谱法测定杨梅中29种农药残留的分析方法,并将本文建立的方法应用于30份市售杨梅样品中农药残留检测。该方法快速、简便、准确和灵敏,适用于理化实验室大批量样品的检测。
